# Gender and Music Composition: A Study of Music, and the Gendering of Meanings

**DOI:** 10.3389/fpsyg.2016.00411

**Published:** 2016-03-31

**Authors:** Desmond C. Sergeant, Evangelos Himonides

**Affiliations:** UCL Institute of Education, University College LondonLondon, UK

**Keywords:** musical gender, gendered music composition, masculinity and music, femininity and music, perception of gendering, sex attribution, MASCFEM

## Abstract

In this study claims that music communicates gendered meanings are considered, and relevant literature is reviewed. We first discuss the nature of meaning in music, and how it is constructed and construed. Examples of statements of gendering in the literature are cited, and the problems identified by writers who have questioned their validity are considered. We examine the concepts underlying terminology that has been used in inconsistent and contradictory ways. Three hypotheses are posed, and tested by means of two listening tasks. Results are presented that indicate that gendering is not inherent in musical structures, but is contributed to the perceptual event by the listener.

## Introduction

### Music and meaning

Composers assemble sound events—pitches, durations, timbres, and silences—to create musical gestures that enact aspects of their inner musical and emotional experiences; the gestures are ordered in sequence to form musical narratives whose purpose is to express and communicate the composer's experiences and ideas to listeners (Ballantine, 1983, p. 2). The sounds are therefore *intentional*, and their intention is to convey *meaning;* “music is therefore a denotive language” (Monelle, 2000, p. 5).

But what is the nature of the meaning conveyed; are there limitations to the ideas and emotions that music can express? Are the meanings intended by the composer the same as those experienced by the listener? If they were not broadly similar, could music be regarded as a language?

Studies by Behrens and Green (1993); Gabrielsson and Juslin (1996); Gabrielsson and Lindstrom (2010); Juslin and Laukka (2003); Juslin and Timmers (2010); Kaminska and Woolf (2000); Quinto et al. (2014); Thompson and Robitaille (1992), and others reassure us of at least a reasonable correspondence between the intentions of purposely composed passages and the meanings attributed to them by listeners. This confirms the view of Monelle (2000, p. 4) that the relation of musical signifier to signified may be imprecise but is not arbitrary: “music is permeated through with intentionality, similar to language, but is not a sign-system” (Adorno, 1998, p. 3).

The problem of meaning in music has been the subject of extended discussion in the literature, and lies within the province of semiotics, the theory of signs and their significations. Semiosis is a process by which a signal becomes a signifier for something else: that which is signified. In the case of music the sounds of the composer's musical gestures are the signifiers, they are symbolic representations of his or her experiences, and the listener must recognize the symbols in order to recover the same experiences—the music's message—from the sounds, a process that requires that composer and listener share a common vocabulary.

A first level of meaning is that of the compositional structures themselves, what has been termed “structural meaning” (Christensen, 1995) “embodied” or “intra-musical meaning” (Meyer, 1956) and “inherent meaning” (Green, 1997a, p. 6), i.e., meaning that is contained within the music itself. These terms refer to the concept of predictive meaning whereby a gesture may lead to expectancy of future gestures having similar or related characteristics, or the recognition of repetition or the elaboration of the features and structure of gestures occurring earlier in the composer's narrative—music referring to itself.

Although this is an important element of communication in music, the meaning of music is not entirely contained in its organization of sounds; it is also dependent on the contribution the perceiver brings to the event. In a non-musical context for example, the sound of a knock at the door would constitute a sign, its signification being that a caller is outside, and the adjustive response of the hearer being that the door is opened. The affective value of the knock, however, would depend on the anticipation of the person opening the door as to the probable identity of the caller: the postman—a lover—a debt-collector—the police! The affective value of the knock might therefore range from pleasure to horror, depending on the contextual expectations the hearer brings to the event.

It has therefore been argued that musical meaning is dependent on social context, that it is “socially and culturally constructed” (Olsson, 2007, p. 989) “a culturally defined artifact” (Lipscomb and Tolchinsky, 2005, p. 384), “growing out of specific social context, and expressing the assumptions of that context” (Citron, 1993, p. 120), “setting life to music” (Ethel Smyth, 1928), telling “the truth about life” (Solie, 1993, p. 10), part of our sense of “the particular structuring of the world implicit in classical music” (Shepherd, 1991, p. 14, 162). DeNora (2008) writes of “The musical composition of social reality.” For Rycenga (1994) “Music is life” (p. 284).

### Music and gendered meaning: a review of the literature

The above reasoning has been carried further to argue that as social meanings are almost invariably gendered, so also are musical meanings. Music has been described as “a dynamic mode of gender” (Taylor, 2012) “an essentially gendered discourse”, “a marker of sexual identity…” meaningful only within a context of “…gender, race and ethnicity” (Treitler, 2011), “fraught with gender-related anxieties,” and the history of musical form and structure described as “a heavily gendered legacy .… bound up with issues of gender”, and that “classical music—no less than pop—is bound up with issues of gender” (McClary, 1991, pp. 16, 18, 54).

The basic structures of music have been held to be gendered (Maus, 1993; Brett et al., 2006), described as “gender-related characteristics of the music itself” (Green, 1997a, p. 139) and as the “gendered meanings of absolute music” (p. 167), “…present in the resolution of chromaticism to the triad”…thereby…“taking on the cultural cast of femininity” (McClary, 1991, p. 124). They have been seen as representing “an image of a gendered hierarchy of political and social hegemony” (Shepherd, 1991), with even the harmonic series is seen as a “social organization of natural phenomena” (p. 98). Hargreaves et al. (2005) list gender, age and nationality as relevant composer-variables in the music-communication process (pp. 15–17).

The contrasting characters of the first and second themes in sonata form have been seen as representing sexual dissonance, the second theme as being “of more tender nature, flexibly rather than emphatically constructed … as it were the feminine to the preceding masculine” This analogy, first made by Marx (1845) in a music-theoretic text has subsequently become a commonplace, as has application of the description *feminine ending* to a cadence that reaches closure on an unaccented beat. The opening of the Tristan prelude has been described as proceeding by “feminine quavers and dotted rhythm” (Monelle, 1995, pp. 103–104) and the chromaticism of the same passage as reflecting “seductive, deadly feminine sexuality” (Clément, 1989); Shepherd (1991) writes of there being “male timbres” and “female timbres” (p. 170) and timbre articulating and reproducing gender identities (p. 160).

The association of music and gendering has been extended beyond gender to sexuality and sexual identity since “musicality is next-door to sexuality” Cusick (1994, p. 71), and “the history of Western music is a history of sexual anxiety, ambivalence, and negotiation” (1993). For McClary (1991), music is “strongly informed by erotic imagery” (p. 124). Female sexual desire and pleasure has been seen as inseparable from musical and devotional experience, even in the convent of Hildegard of Bingen (Holsinger, 2001, pp. 87–181).

The imputed gendering of musical signs becomes even more complex when the connection is argued to extend beyond the boundaries of male/female heterosexuality to sexual orientation. Cusick (1994) describes the effects of “a Lesbian relationship with music” (pp. 67–83) and a “lesbian reception of music's message” (p. 67). Rycenga (1994) discusses “lesbian compositional processes” (p. 275), stating that being a lesbian “transforms the thought/action process that is composition.” Strong associations have been identified between Tchaikowsky's fourth symphony, Opus 36, and the composer's reported homosexuality (Jackson, 1995; Brown and Abraham, 1997).

But by what signifiers could gender or sexuality be communicated by the sounds of music? It has been suggested that transmission is achieved by codes embedded in musical gestures and thus conditioning their message. McClary (1991) for example, writes of “the common semiotic codes of European classical music: the gestures that stereotypically signify ‘masculinity’ and ‘femininity’” (p. 68), and says (p. 8) that “these codes change over time” because “the meaning of femininity was not the same in the eighteenth century as in the late nineteenth, and musical characterizations differ accordingly” and so the codes “are informed by the prevalent attitudes of their time,” but are nevertheless “strikingly resilient.”

Solie (1993) says of the music of the composer Ethel Smyth that “her own lesbian experience—her difference—may be encoded in her instrumental music” (p. 10). Wood (1995) agrees, saying that Smyth used music “in ways that simultaneously reveal and conceal her lesbian experience,” and that “it was fugue's metaphorical association with sexual seduction that suggested ways in which to reconstruct her erotic relationships with women” (p. 166). Rycenga (1994) proposes that “Music is erotic because of culturally encoded signals” (p. 284). McClary (1991) says of Tchaikowsky “As a composer, he inherited a code of signification that marked themes as either masculine or feminine” (p. 78), though in referring to the composer's fourth symphony she acknowledges the possibility of some ambiguity in the codes “This is not to suggest that the piece should be understood only as the narrative of a homosexual male” (p. 77). Green (1997a) sees a direct association between gendered information inherent in the composition and the gender of the composer: “music can delineate a notion of femininity or masculinity owing to the gender of the composer” (p.131). The essential notion inherent in the statements of Green and McClary is that the gestures embedded in the narratives of compositions by women composers impart information that is qualitatively different from those that characterize the narratives of male composers.

Juslin (2001, p. 324) talks of the presence of cues rather than codes, suggesting that these might devolve on factors such as tempo, dynamics, timing and articulation, (of which tempo and dynamics appear to be the prime agents (Kellaris and Kent, 1991; Juslin and Madison, 1999; Webster and Weir, 2005; Gomez and Danuser, 2007; Sergeant and Himonides, 2014), but using a *lens* model (Brunswick, 1956) he argues that these are used interactively and on a probabilistic basis, and are therefore approximate in meaning, and not representing a systematic lexicon.

Whist recognizing music as being a “denotive” language, Monelle (2000) bluntly dismisses the possibility of the kind of gendered meanings for music attributed by the many writers cited above, saying that “Music does not signify society. It does not signify literature. And most of all it does not signify ‘reality’” (p. 19), and that if music codes exist at all, they are essentially intra-musical: “codes are proper to music” (p.19). Davies (1994) takes a similar view that “talk of a syntax and vocabulary for music is best avoided” (p. 3). Attempts to identify a specific lexicon of musical meanings, such as that of Cooke (1959) have largely failed to convince.

The weakness of assertions of music's gendering is that its claimants have consistently failed to explain how codes of gendered communication might function (Biddlecombe, 1992). Without a clear account of the musical means by which composers imbue their works with gendered information, with correlating demonstration of the reception of that information by listeners, the claims must remain at the level of metaphor, and basic questions remain about what is communicated by the structures and sounds of music. As Mendelssohn remarked in a letter of 1842, “There is so much talk about music, and yet so little is said” (Mendelssohn-Batholdy, 1842).

Is there an identifiable “woman's voice” in composition, a quality of *woman-composer-speak*, “a specifically female style in music” (Citron, 1993, p. 11), and if so, in what features of musical structure, gesture, implication, or affect does it differ from *man-composer-speak*, and by what differences of musical structure might a listener distinguish between a composer whose sexual orientation is heterosexual and one who is lesbian or gay? What particular conformations of musical gestures would mark themes as either masculine or feminine? Propositions of gendering raise basic questions about what meanings musical compositions can express or reflect of the people who make and use them (Solie, 1993, p. 3).

The proposition that creative output is gender-specific is negated by the frequency with which opposite-sex identities have been adopted by authors and composers for purposes of publication. In the field of literature this has been a common practice: the early poems of the Brontë sisters, Charlotte, Anne, and Emily were published under the fictional names of Currer, Acton, and Ellis Bell respectively^1^; Mary Ann Evans adopted the *nom-de-plume* George Eliot; Louisa May Alcott wrote as A.M. Barnard; other examples include Amantine Dupin (Georges Sand), Nellie Harper Lee (Harper Lee), Violet Paget (Vernon Lee), Karen Blixen (Isak Dinesen), Alice Sheldon (James Tiptree), and Marie Bobillier (respected music historian Michel Brenet). Others have elected for the ambiguity of initials, for example P. D. James, and J. K. Rowling. Male adoption of female identity has been less frequent, though it is not unknown: the romantic novels and poems of Fiona Macleod, for example, were written by William Sharp (Halloran, 2015). Similarly, it was fairly common practice in the eighteenth and nineteenth centuries for music of women composers to be published under male names (Citron, 1987, p. 113), sometimes those of established male composers, or for a teacher to include works of a student among his own publications (Jackson, 1991). The works of Isabella Leonarda (1620–1704) thus appear in a collection by Gasparo Casati *maestro di capella* of Novara Cathedral. Early songs of Josephine Lang were published by Robert Schumann (Krebbs and Krebbs, 2007), compositions of Maria Anna Mozart (Nannerl) were published for her by her more famous brother under his name (Mozart's Letters, July 7, 1770). Six of the songs published as works of Felix Mendelssohn, Opuses 8 and 9, were in fact composed by his sister Fanny (Kőhler, 1980; Reich, 1991; Tarpenning, 1997, p. 30; Gates, 2007), and according to Hoffer (2015); Hoffer and Bailey (2016) an undetermined number of her other works also appeared under her brother's name; Citron (1987) reports that none of Fanny's works were published in her own name until 2 years before her death. Whatever the motive for the adopted sex of authorship, the agreement of both actual and nominee composers to publication in this way indicates that neither considered the possibility of the presence of any disclosing gendered markers in the listening experience of their music.

Halstead (1997, p. 215) addresses these problems and raises the following questions:

- Is either the sex or gender of a composer evident from the musical content of a composition?
- Do the sounds in a composition, or their organization communicate information relating to sex or gender?
- If music is perceived by listeners as being gendered, is the extent or direction of the gendering influenced by the sex of the listener?

### Sex, gender, and gendering: an analysis of underlying concepts

The review above illustrates the confusion that has that has prevailed in use of terms relating to gender and sexuality and the consequential confusion of their underlying concepts and inferences. Their meanings and conceptual bases are therefore examined below.

#### Sex, sexuality, and sexual orientation

**Figure 1:**
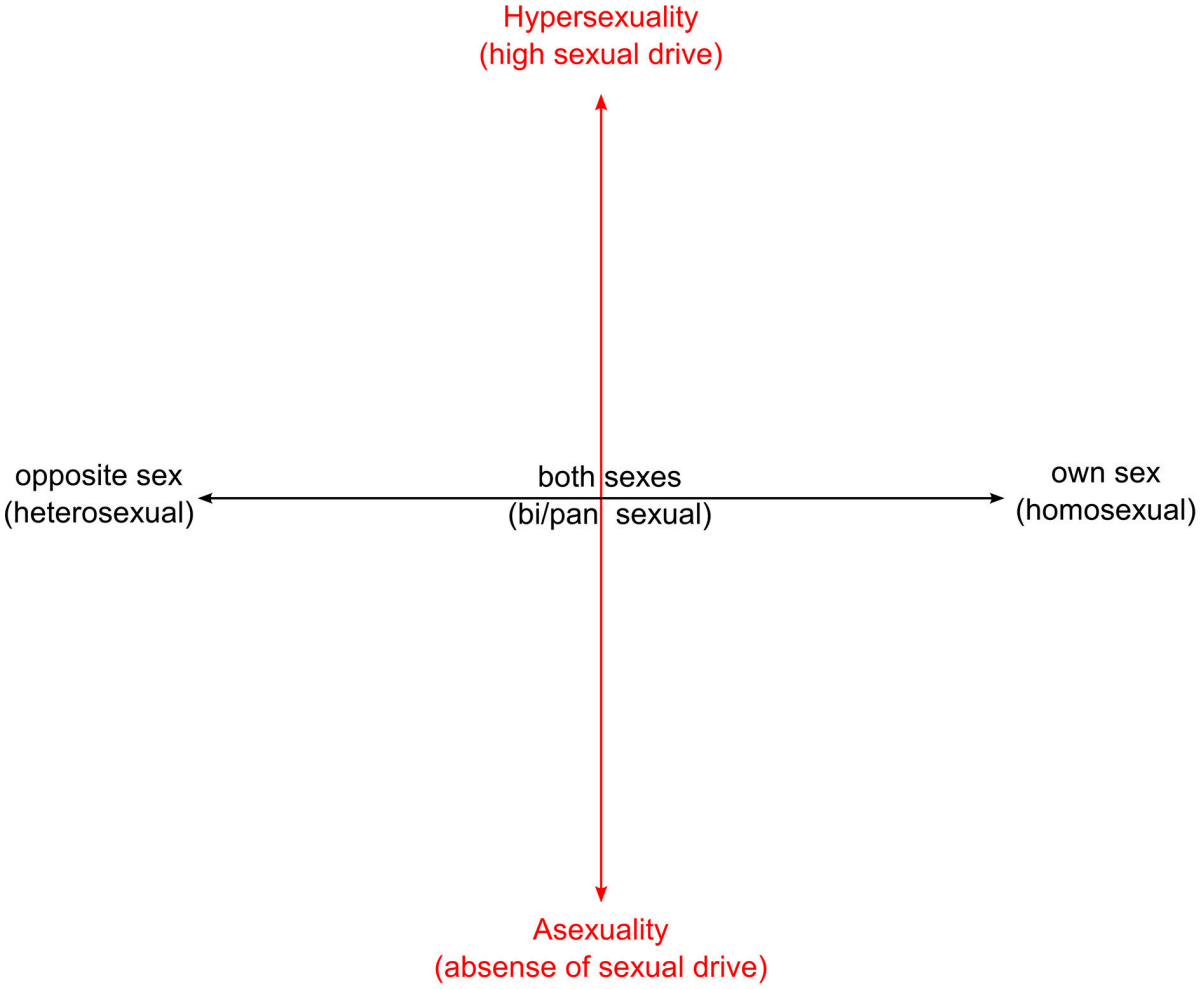
**Schematic model of sexual orientation and sexuality**.

Sex is biological and dichotomous: except in the small number of cases of genetic irregularity or surgical intervention, humans fall into one of the two discrete biological categories male or female. There are no scales or degrees of sexing. Sexual orientation and sexuality constitute two separate parameters of persona (Melby, 2005; Prause and Graham, 2007). Sexuality refers to the capacity of humans to experience erotic feelings and responses as a consequence of interactions with others. Degrees of arousal can be conceived as varying from asexuality (lack of sexual drive or arousal toward other persons whatever their sexual orientation) to hypersexuality, where sexual drive becomes obsessive (Bogaert, 2006; Prause and Graham, 2007; Figure 1).

Sexual orientation can vary through a continuum that ranges from heterosexuality/straight (sexual or romantic attraction exclusively to the opposite sex), through bisexuality/pansexuality (attraction to both sexes), to homosexuality/lesbian or gay (attraction to same sex), (American Psychological Association, 2011).

#### Gender and gendering

**Figure 2:**
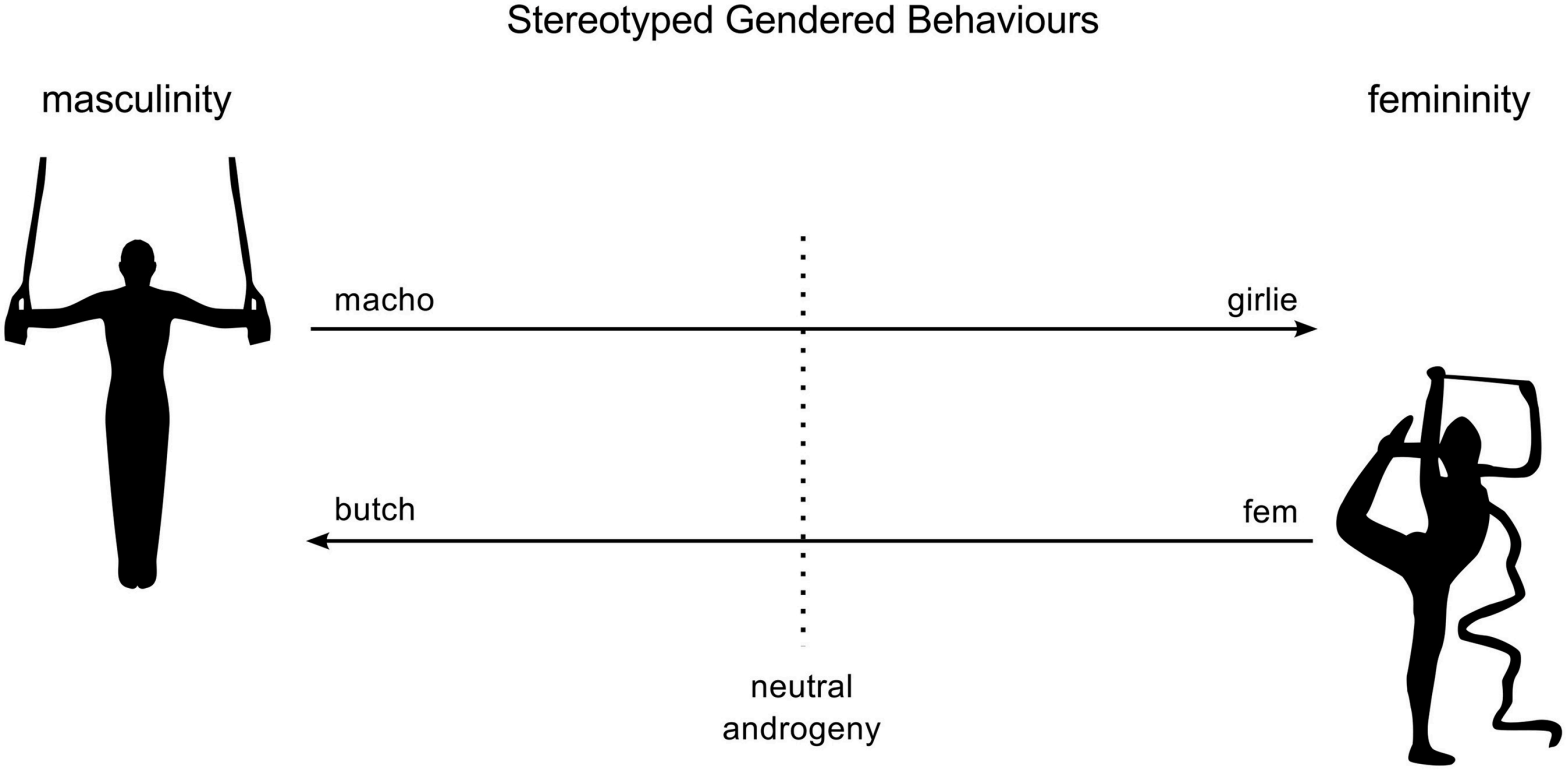
**Directional trends of gendered behaviors**.

Gender refers to a complex concatenation of behaviors which include adopted speech and dress styles, preferred communicative modes, personal associations, social roles, attitudes, self-concept, and social values, all of which are shaped by environmental forces. Behaviors of males can extend from those perceived to be characteristic of extreme masculinity to those deemed typically feminine; behaviors of females may, conversely, range from those that are perceived as characteristically female to those that are regarded as typically male (Figure 2). As Keener (2015) points out, gender is a highly complex concept.

Masculinity and femininity are not simple unidimensional categories; rather “they are loosely constructed categories that contain a variety of characteristics” (Deaux, 1987). Masculinity and femininity are better seen as contrasting polarities of two continua of complex behavioral tendencies. The relative distances between these polarities are subject to change in parallel with changes of societal values and behaviors (Twenge, 1997; Figure 2).

Gendering is the attribution of qualities or behaviors typical of either masculinity or femininity to persons, objects or phenomena: “the ascription of masculine or feminine quality to some pair of opposed entities or concepts” (Guck, 1994). Gendering is therefore not an inherent quality of an object, but is a comparative ascription to it, functioning as a form of simile. An object cannot validly be described as gendered unless a direction (male < > female) and a degree (slight < > extreme) on a scale of *masculinity—femininity* is identified for it.

#### Gendering of music

If the information conveyed by musical compositions can be considered to be gendered (c.f. the statements of Cusick, Hargreaves, McClary, Rycenga, Taylor, Treitler, and others, cited above) this indicates a belief that gestures and structures employed within a composition possess direct relationships to traits, qualities or behaviors perceived to be characteristic of either the masculine or feminine directions of gender. But given that scales of are not discrete, but are inherently reciprocal (Figure 2), a position proposed for a composition on either scale could therefore equally well be conceived as occupying an equivalent position on the opposite scale. The notion of gendering of a composition or passage then looks decidedly hypothetical.

#### Gendering of listeners

It has been suggested that perception of gendering of a musical composition may be dependent on the sex of the listener, which may “influence their overall response to music, and perception of masculinity or femininity, so that … men and women must have a slightly different type of musical experience resulting from their gender” (Green, 1997a, p. 135). Green's statement indicates that gendering is not an inherent property of the music, but is imposed on it in the listener's perception. This proposition has subsequently been elaborated by Bem (1981, 1987, 1993) through her Gender-Schema Theory under which she argues that gendering of phenomena is attributable to a human propensity to process and categorize information in gender-related, sex-typical ways. Attributes and behaviors are sorted into masculine and feminine categories or “equivalence classes” regardless of their differences on dimensions unrelated to gender. Bem argues that the tendency for sex typing has its origination in the self-concept, and particularly the self-sex-typing of the perceiver.

#### Gender stereotypes

Gender stereotypes have sometimes been regarded as negative, delimiting, demeaning, and in conflict with human rights (Cook and Cusack, 1979; United Nations, 1979). In recent psychological literature, however, they have been seen more objectively as culturally sourced cognitive systems of categorization into broad male/female groupings based on the relative presence or absence of specific features and personality traits. Gender stereotypes are not only descriptive of how men and women typically are, they tend also to be prescriptive, indicating socially preferred characteristics, that is, how men and women should be (Eagly and Steffen, 1984; Schein, 2001; Heilman and Wallen, 2004; Perez-Quintana and Hormiga, 2015). The categories have been described as being organized on a probability of occurrence of prominent traits and characteristics (Deaux and Lewis, 1984; Gupta and Bhawe, 2007). As an example, in language production, women have been shown to employ higher and wider frequency ranges and more frequent pitch excursions than do men (Harries et al., 1998; Sergeant, in press). Listeners use these acoustic properties of voice in making gendered attributions to speakers (Apple et al., 1979). Vocal pitch and style are thus perceived as indicative of masculinity/femininity and sexual orientation (Gaudio, 1994; Smyth et al., 2003). Men whose speech habitually employs above-modal frequencies and modulatory patterns are perceived to occupy positions toward the feminine polarity of gendered behaviors. Higher male voices have been perceived as more submissive, less truthful, less persuasive (Apple et al., 1979; Huron et al., 2006) but lower pitches as representing authority, confidence, aggression, and dominance (Scherer et al., 1973; Morton, 2006; Puts et al., 2007; Dey et al., 2012).

Stereotypical words associated with femininity include: *emotional, sensitive to feelings of others, expressive, submissive, nurturing*: those associated with masculinity are *aggressive, dominant, competitive, agentic*. The respective adjectives have been summarized succinctly as “feminine = expressiveness, male = instrumentality” or “agency versus communion” (Spence and Helmreich, 1980; Lippa, 2001; Schmader and Block, 2015).

Gender stereotypes form the basis for gendering of objects or phenomena which thus takes on an adjectival function. Unger and Crawford (1996) present an extended review of the ways in which gendering and gender stereotyping are imposed by societal forces, beginning even before the birth of a child, and subsequently through differentiated patterns of communication, physical contact, clothes and toys. Parental attitudes thereby come to permeate the child's own attitudes, preferences and perceptions of gendering of attributes.

Abeles and Porter (1978) have clearly illustrated the presence of stereotyping in music; in their study they asked parents to identify the instruments they would most prefer their children to learn; choices for boys were drums, trombone, and trumpet, but for girls flute, clarinet, and violin, these choices reflecting stereotypical male characteristics of robustness and dominance, but sensitivity, expressiveness, and submissiveness of females. Unsurprisingly, subsequent studies by Delzell and Leppla (1992); O'Neill and Boulton (1995, 1996) have shown children's own choices of instruments for the two sexes clearly reflect those of parents, and adults and children of both sexes have been shown to share these gender-stereotypical perceptions, (Griswold and Chroback, 1981).

#### Gender identity

Gender identity or self-sex-concept refers to a person's perception of his/her own characteristics in relation to population scales and norms of gender traits, and may unconsciously operate as a lens through which gendered characteristics in objects and other persons are judged (Wood and Eagly, 2015). For example, Fraccaro et al. (2011); Feinberg et al. (2005, 2011) and Vukovic et al., 2010) have all shown that female preferences for male voice pitch are associated with their own voice pitch.

### Qualitative differences of musical thought and creativity in compositional output of male and female composers

The historical/societal reasons for the relatively small number of women composers over successive eras have been widely rehearsed in recent literature (e.g., Citron, 1993; O'Neill, 1997; Andrew, 2012; Hewitt, 2014; Hoffer, 2015, p. 197) the broad view is that there are no intrinsic grounds for a belief that music by male composers is qualitatively different or superior to that composed by women or vice-versa. Quality of an artistic product is indefinable and unquantifiable and putative superiority of creativity or creative musical output of either men or women is not a concern of this study.

### Hypotheses and statistical decisions

The present study tests three hypotheses:

1. The sex or gender of its composer is identifiable from the musical content of a composition;
2. perception of gendering of music is related to the sex of the listener;
3. musical sounds, or the organization of sounds within a composition, infer sex, or gender characteristics.

Necessary conditions for support of these hypotheses are:

- That the sex of composers be accurately and reliably identified by listeners, at a level above chance, from a broad sample of art music;
- a consistent direction of gendering (i.e., toward either masculinity or femininity) should be reported for each composition at normally accepted levels of reliability and significance;
- gendering should be evident for all compositions, whatever their era or style of composition. Gendering of only a few compositions would fail to indicate a general principle of the presence in music of masculine or feminine characteristics;
- in the case of Hypothesis 2, composer-sex attributions for works by male composers would be reliably differentiated from those for female-composed works by sex of listener.

## Materials and methods

### Materials

#### Listening sequence

A listening sequence was constructed as a data-collection instrument: this comprised 36 extracts taken from published recordings of music of the Western art/classical repertoire. Eighteen of the extracts were taken from works by female composers, 18 from works by male composers.

The eras of composition of the music selected ranged from mid-seventeenth century to late twentieth century, and works were chosen so as to represent the main genres of musical composition, i.e., works for full orchestra (symphonies or concerti), works for smaller orchestral or choral groups, chamber music (trios or quartets), works for solo keyboard, or single voice or instrument with accompaniment. Selection was made so that sampling of features such as tempi and major-minor modalities, etc. was equally balanced between composer-sexes, for example, the same number of extracts from female-composed symphonic works was included as from those that were male-composed. It was obviously necessary that while ensuring that the extracts chosen provided a representative sample of art music, works that would be immediately recognized by listeners, would have to be avoided. The compositions selected (Appendix A in Supplementary Material) were therefore not by composers who could be regarded as obscure, but were compositions that receive less frequent performances in concert programmes and were therefore unlikely to be recognized by listeners.

The number of performers participating in the performance of the music of each extract was estimated. In cases of solo works, duos, trios, and quartets this number was self-evident, but in others, for example choral or orchestral works or concerti, the number of performers was estimated from the instrumentation of the score, current orchestral membership lists, and the apparent tonal density of the recording. This provided an order of works by tonal density: the greater the number of musicians contributing to the performance, the greater the tonal density created by multiple instrumental sources. An obviously related variable was an order of scale of work: short solo to symphony.

Extracts were of approximately 1.5 min duration taken from either the opening passages of the compositions, or subsequent representative points in their progress, ending at convenient musically appropriate points. In cases of some shorter works the entire composition was heard within this duration and in order to maintain equality of listener-exposure among extracts, all 36 extracts were of the same length. The total duration of the listening sequence was thus 54 min, plus short intervals for moving between extracts; this was judged to be the maximum duration that volunteer listeners would be likely to persevere with the task to completion.

The listening sequence was made available to listeners by means of an online facility on the web server of the International Music Education Research Centre (iMerc) at the then Institute of Education (since December 2015, a faculty of University College London). When potential listeners logged on to the dedicated website, they were presented with an introductory page of information explaining the background, motives, and procedures of the research. When they had read to the end of the introduction, they were asked to indicate whether they wished to participate in the study: on an affirmative response, the system led them directly to a page of *Instructions to listeners* and then, on an indication that they were ready to proceed, to the first extract of the sequence.

### Methods

#### Participants

The nature of the listening material required that participants should have extended familiarity with idioms of art music across successive eras of composition, and be sensitive to subtle nuances of musical gestures and structures. Volunteers were accordingly sought from an international constituency via music research centers, instrumental groups and music societies.

Ninety two unpaid volunteers, 37 male, 55 female, with an age-range of range 18 to 85 years and mean age 40 years 11 months acted as listeners; the sample included professional instrumentalists, music scholars and researchers, and musically competent amateurs. Data from all listeners who provided responses to all 36 extracts were included in the database. Seven persons who initially logged on to the system completed only the first few extracts, and the small amount of data they contributed was excluded.

#### Procedure

Listeners undertook one of the two separate tasks: no listener undertook both tasks. Seventy one listeners (44 female, 27 male) undertook the *composer-sex attribution task.* 21 listeners (10 male, 11 female) were assigned to the *music characteristics ratings task.* The two resultant databases were thus independent and there was no possibility of cross-contamination.

#### Composer-sex attribution task

At each extract in the listening sequence, listeners were asked to judge whether the composer of the work being heard was female or male. They were then asked to qualify their *male/female* response by rating their confidence in their decision using a 7–point scale where 1 = *I'm not at all confident* and 7 = *I am very confident*. The procedure was self-pacing: after responding to an extract, listeners were able to move to the following extract when they were ready. Provision was made for the listener to suspend the listening session for resumption at a later time; when they resumed the task, the system restored them to the sequence at the extract following the point of suspension.

An additional response box was provided for a listener to indicate if they recognized the composition and were therefore aware of the identity of the composer. Listeners were given no guidance as to the proportions of male- or female-composed works in the sequence.

In order to avoid possible effects arising from the order in which the extracts were heard, the order of presentation of the 36 extracts within the sequence was randomized into a unique order for each listener.

**Figure 3:**
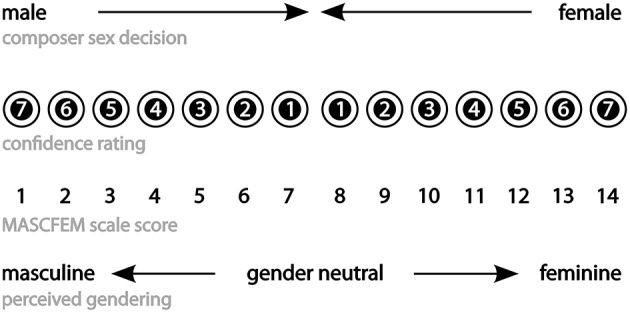
**Structure of the MASCFEM scale: perceived masculinity/femininity of extracts and performance**.

A sensitive measure—the MASCFEM scale—was constructed from the data generated by the composer-sex task by combining *male/female* composer-sex decisions with their associated confidence ratings. This provided a 14–point measure of perceived masculinity/femininity of the musical extracts. A decision that a composer was *male* made with maximum confidence rating of 7 was taken as the optimal masculine polarity of the scale resulting in a low MASCFEM score = 1; confidence ratings ranging downwards through 6 to 1 were taken to reflect a progressive reduction in masculine information. A response of *female* made with a confidence rating of 7 was taken as the optimal point of femininity giving a high MASCFEM score = 14. The mid-point of the scale (scores 7–8) thus represented near gender-neutrality (Figure 3).

#### Music characteristics rating task

**Figure 4:**
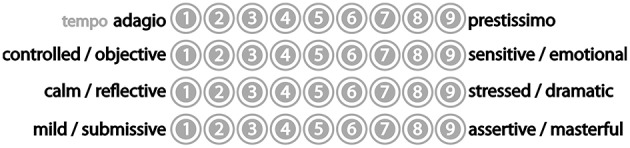
**Four music characteristics scales**.

The separate group of 21 listeners heard the same sequence of extracts, but were asked to rate each extract on four 9–point semantic differential scales whose verbal polarities were designed to provide measures of the perceived tempo, emotional valence and character of each extract (Figure 4).

Data from the composer-sex identification task (described below) will indicate whether the sex of the composer is discernible in the compositional structures of the musical extracts, i.e., will test the postulated presence of reliable differences between products of male and female composers. The statistical decision will be resolved on the probabilities of accurate composer-sex identifications. Data from the MASCFEM scale will determine whether the information communicated by the composition is perceived as gendered to satisfactory levels of reliability, and indicate the direction of any such gendering.

The possibility was recognized that data obtained from the composer-sex attribution task might unintentionally be influenced by the particular selection of compositions from which the extracts were drawn. It has previously been reported that masculine or feminine associations may arise from music when a combination of characteristics of musical valence are present. For example, an impression of masculinity may be gained with faster tempi, but music at slower tempi may be perceived as feminine (Kellaris and Rice, 1993; Horn and Costa-Giomi, 2011; Sergeant and Himonides, 2014). *T*-tests were therefore applied to data from the Music Characteristics listeners to ensure that no differences were present between female-and male-composed extracts: all tests proved non-significant, confirming that in these respects no such influences could cause bias in data of the composer-sex attribution.

#### Ethical issues

The research was conducted in line with the American Psychological Association (APA) Ethical Principles of Psychologists and Code of Conduct (effective June 2010). This research was not required to undergo an evaluation from University College London (UCL) Research Ethics Committee, as it entails Artistic Criticism, a focus that is exempt from the Committee's remit (see: http://ethics.grad.ucl.ac.uk/exemptions.php). Regardless, all research participants were consenting adults who were made aware that they could withdraw at any point of their engagement with the online response form (s). Additionally, the online response forms were designed so that it is impossible for any individual to be identified from the recorded response data.

## Results

### Hypothesis 1. statistical considerations: composer-sex identification task

With 36 extracts in the listening sequence, and a two-alternative composer-sex response of male or female, the inherent probability of the composer-sex measure was *p* = .5, giving a predicted score at the binomial mean of 18, with a standard deviation of 3. The range of scores that could be regarded as non-significant variants of the binomial mean would therefore notionally extend between ± 1.96 σ (i.e., the point where *p* = 0.05 probability would commence), here ranging from a minimum of 12.22 to a maximum of 23.88. Scores significantly removed from the mean at a probability of *p* < 0.05 or smaller would therefore be 12 and below or 24 and above.

The overall mean score obtained for correct attribution of sex of composers of the 36 extracts by our 71 listeners was 15.34, with σ = 3.29, i.e., below the binomial mean. Neither sex of listener showed superiority in composer-sex-attribution (mean for female listeners = 15.68, male listeners 14.78, [*t*_(69)_ = 1.12, *p* = 0.265, difference not significant]. Only one listener obtained a score exceeding the point of 1.96 standard deviations from the expected mean, recording 28 correct attributions. Given a population of *n* = 71 scores this single listener would be within the number of above-chance scores within the distribution that could be expected by chance, and his performance may therefore not be interpreted as indicating presence of reliable ability to correctly attribute the sex of a composer. We therefore must conclude that the listeners of our musically experienced sample were not able to determine the sex of composers from examples of their work

#### Distribution of composer-sex attributions

Listeners made significantly more male-composer attributions than they did female: of the total 2, 556 listener responses, 1687 (66%) attributed extracts to male composers, 869 (34%) to female composers, (mean attributions per extract to male composers = 23.76, to female composers = 12.24, [*t*_(70)_ = 11.268, difference significant *p* < 0.000]. One listener attributed all 36 extracts to male composers; 24% of listeners made 10 or fewer female attributions. This bias toward male attributions was evident equally in the judgments of both male and female listeners [*t*_(69)_ = 0.308, *p* = 0.759, difference not significant]. Responses of listeners did not differ by category of musical occupation (full-or part-time professional, competent amateur, etc.) or listener age.

In all cases where *t*-tests were applied, requirements for homogeneity of variance were met.

#### Recognition of extracts

Of the total 2556 responses there were only 36 instances of listeners reporting that the music of an extract had been recognized and the sex of the composer was therefore known to them; more than half of these claims were attributable to just two listeners. In 19 of the 36 instances the composer's sex had been identified incorrectly, indicating that the extract had not in fact been recognized as claimed, the composition presumably having been confused with another of the same musical era. The possibility that familiarity with the works included in the listening sequence might affect data was therefore discounted.

#### Ensemble size and perception of masculinity

**Figure 5:**
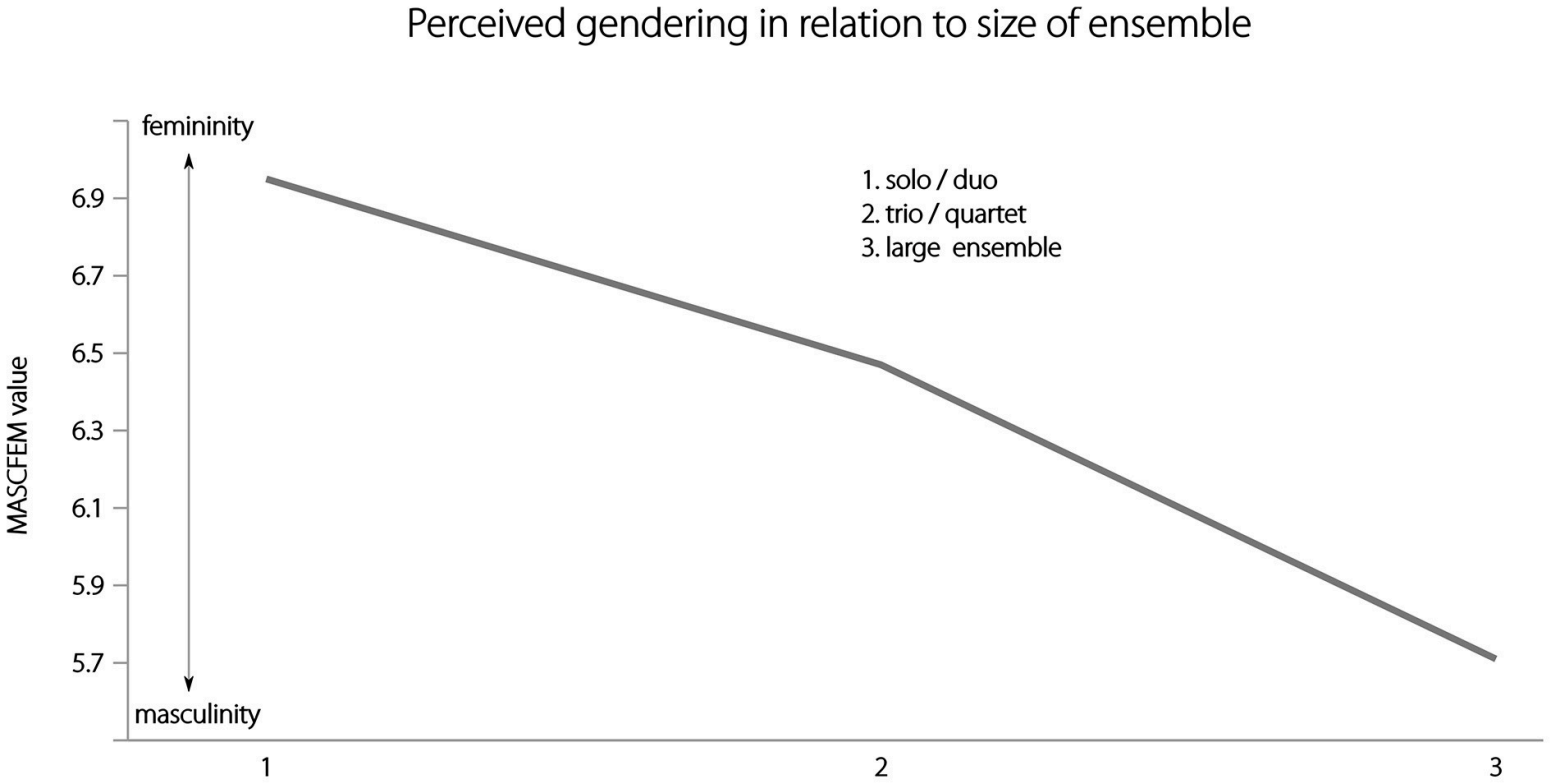
**Increase in perceived masculinity with increasing size of ensemble**.

Composer-sex attributions were found to correlate significantly with the estimated number of participating musicians (*r* = 0.470, *p* < 0.000): the greater the number of musicians taking part in the performance of the work, and therefore the greater the tonal density created by multiple instrumental sources, the more likely it was that the music would be perceived as the work of a male composer. When the scale of the work (i.e., solo, duo, chamber work, work for small orchestra, full orchestra) was used as an alternative comparator with composer-sex attributions, the magnitude factor tendency to male-composer attributions for works of larger scale of work increased (*r* = 0.534, *p* < 0.000; Figure 5).

#### Major and minor modalities

MASCFEM values attributed for works in major mode were significantly higher than for those in minor, i.e., works in major keys were judged more feminine than those in minor keys, which were judged more masculine [*t*_(70)_ = 6.078, *p* < 0.000 and *r* = 0.432 *p* < 0.000].

### Hypothesis 2. direction of attributions

One of the criteria set as evidence of gendering of extracts was that there should be satisfactory consistency of gendering for extracts, i.e., directionally toward male or female. A binomial test was run using the total of sex-attributions for each extract to determine whether a clear direction was evident. For 15 of the 36 extracts no statistically significant direction was present. Results showed that the bias toward male-composer attributions prevented such consistency (Table 1).

**Table 1:** **Directions of composer-sex attributions**.

**Composer sex**	**Attributed sex**	**Total extracts**
Male	Male	12
Male	Female	5
Female	Female	3
Female	Male	15

No unanimous level or direction of gendering was present for any extract: where there was near-unanimity among listeners, this was traceable to influences such as the magnitude factor of larger instrumental forces with consequent greater tonal density, as for example, with symphonic works or concerti (for example: Louise Farrenc Symphony = 89% direction male; Clara Schumann Piano Concerto = 90% direction male; William Sterndale Bennett Piano Concerto = 89% direction male). The required condition of consistency of direction as evidence of gendering was therefore not met.

#### MASCFEM scale

MASCFEM (masculinity-femininity) scale values for extracts were examined by *t*-test with sex-of-composer as the grouping variable. A highly significant difference was found [*t*_(70)_ = 4.531, *p* < 0.000] with music of male composers showing higher values (i.e., greater perceived femininity) than did works composed by females.

When MASCFEM values for male- and-female-composed extracts were examined with sex-of-listener as the grouping variable, this trend in perceived masculinity/femininity was found to be present equally in the judgments of both male and female listeners [values for works of male composers *t*_(69)_ = 0.233, *p* = 0.816, for works of female composers *t*_(69)_ = 1.589, *p* = 0.117, both differences not significant). The suggestion that perception of gendering is related to sex of listener was therefore not supported.

### Hypothesis 3: gendering and music structure

#### Music characteristics ratings

Assurance that listeners assigned to the music characteristics task showed a satisfactory level of agreement was essential for validity of the four scales. Ratings of the 21 listeners on the four music characteristics scales showed remarkable consistency, with a mean inter-listener correlation of *r* = 0.417, *p* < 0.000. Of the 420 inter-listener correlations, only 18 failed to reach significance at *p* = 0.05 level, and the majority were significant *p* < 0.001. These results were therefore taken as validation of the four scales as effective measures of musical affect and meaningful descriptors of real musical valence.

No differences between male and female listeners in their application of the four Music Characteristics scales were found: ratings on all four scales yielded non-significant *t* ratio values.

#### Correlations among four music characteristics scales

The four scales were shown to be inter-related (Table 2), primarily through the influence of tempo as a determiner of musical valance and mood (consonant with findings of Hevner, 1937; Rigg, 1940; Holbrook and Anand, 1990; Kellaris and Kent, 1991; Husain et al., 2002; Horn and Costa-Giomi, 2011 and previous evidence of the present authors, 2014).

**Table 2:** **Correlations between Music Characteristic Scales**.

	**Controlled/objective—sensitive/emotional**	**Calm/reflective–stressed/dramatic**	**Mild/submissive–assertive/masterful**
Tempo adagio–prestissimo	–0.621^**^	−0.421^*^	0.706^**^
Controlled/objective—sensitive/emotional		−0.094	0.309
Calm/reflective–stressed/dramatic			−0.746^**^

** p < 0.000;

* p < 0.010.

Interpretations of these correlations are that:

Tempo is powerfully related to all three other scales:

Faster tempi brings more controlled objective, stressed/dramatic, assertive/masterful
Slower tempi brings more sensitive/emotional, calm/reflective, more mild/submissive
More stressed/dramatic is associated with assertive/masterful Controlled/objective–sensitive/emotional scale is related to tempo, but not to the other two scales.

### Musical valence of extracts: MASCFEM values and music characteristics ratings

MASCFEM values for extracts were found to correlate significantly with ratings for three of the music characteristics scales (Table 3).

**Table 3:** **Correlations of MASCFEM values with music characteristics ratings**.

**Music characteristic scales**	**MASCFEM values**		**Interpretation**
	***r***	***p***	
Tempo: *(Adagio—prestissimo)*	−0.500	< 0.002	The faster the tempo the music was rated by the Music Characteristics listeners, the more masculine it was judged to be by Composer-sex listeners [*t*_(33)_ = 14.62, *p* < 0.000]. Conversely, slower = more feminine
Calm/reflective—Stressed/dramatic	−0.612	< 0.000	The more sensitive/dramatic the music was rated, the more masculine it was judged to be by Composer-sex listeners. Conversely, more calm/reflective = more feminine.
Mild/submissive—Assertive/masterful	−0.693	< 0.000	The more assertive/masterful the music was rated, the more masculine it was judged to be by Composer-sex listeners. Conversely, more mild/submissive = more feminine
Controlled/Objective—Sensitive/emotional	0.612	< 0.000	The more sensitive/emotional the music was rated, the more feminine it was judged to be by Composer-sex listeners. Conversely, more controlled/objective = more masculine

## Discussion

### Hypothesis 1. (the sex or gender of its composer is identifiable from the musical content of a composition)

The poor performance of our listeners in the Composer-sex attribution task (mean score = 15.34, near one standard deviation below the expected binomial mean) indicates that our 71 musically cognizant listeners failed to retrieve gendered information from the musical extracts they heard such as would have enabled differentiation of compositions of women composers from those of men. Data from the MASCFEM scale indicated, counter-intuitively, a significant tendency for works of male composers to be perceived as more feminine than those of women, and conversely, those of women composers to be judged more masculine than those of men.

If the information derived from music provides no clues as to the sex of its composer, it is highly improbable that it will provide any indication as to a composer's sexual orientation or sexuality. Claims such as Rycenga's (1994) that “being a lesbian makes a difference” (p. 275) may therefore reflect a genuine subjective sensation or feeling of self-awareness experienced by the composer during the process of composition, but that experience is not represented in the gestures, structures, or narratives of the music composed.

The first hypothesis, that the sex of its composer is identifiable from the musical content of a composition must therefore be rejected. No differences in the compositional quality of male- or-female-composed works were evident to our listeners: there were no characteristic *musical watermarks* that revealed the sex of a composer or evidence of distinctive musical male-or-female-speak. This accords with the view of McClary (1991) “I do not believe that one can discern a composer's sexual orientation (or gender or ethnicity) merely by listening to the music” (p. 206), dispelling the essentialist notion of “the existence of any specifically female style” (Citron, 1993, p. 11).

### Hypothesis 2. (perception of gendering of music is related to the sex of the listener)

Neither sex of listener showed superiority in composer-sex-attribution, and no inter-relation was found between sex of composer and sex of listener: MASCFEM values showed that male and female listeners responded with near identical composer-sex attributions to male-and-female-composed works (*p* = 0.816). Similarly, no differences were evident in the application of the four Music Characteristics scales in their application by male and female listeners. The second hypothesis, that sensitivity to gender information in music is dependent on or related to the sex of the listener, is therefore also rejected. Female listeners cannot be regarded as having intuitive awareness of feminine elements in the message of works composed by women through commonality of sex, nor do male listeners have gender-privileged insights into male-composed works.

### Hypothesis 3. musical sounds, or the organization of sounds within a composition, infer sex or gender characteristics

Notwithstanding the poor levels of accuracy in the composer-sex attribution task, analysis of responses revealed that they were neither arbitrary nor random. Comparison of composer-sex attributions and MASCFEM values with the reports of listeners in the independent Music Characteristics group revealed systematic patterns in listener responses, and these enabled factors that informed composer-sex decisions to be identified.

Responses revealed a prevailing assumption that musical composition is predominantly a male activity (attributions to male composer = 66%, female-composer 34%, *p* < 0.000). One listener attributed all 36 extracts to male composers; 24% of listeners made 10 or fewer female attributions. Only five listeners (7%) attributed more than half of the extracts to women composers. This bias toward male attributions was equally evident in the judgments of both male and female listeners [*t*_(69)_ = 0.308, *p* = 0.759, difference not significant).

When MASCFEM scores were compared with correlations among Music Characteristics ratings, the results reported above showed composer-sex attributions to have been referenced to an interactive network of representations of masculinity and femininity in music, indicating the presence of a complex schema of inter-related properties. Assembling together the data presented in the Results Section, reveals how these affect perception of musical characteristics (Table 4).

**Table 4:** **Properties influencing attributions of compositions to male or female composers**.

**Maleness**	**Femaleness**	**Probability**
Faster tempi	Slower tempi	< 0.002
Major mode	Minor mode	< 0.000
Greater tonal weight/texture (many performers)	Lighter tonal weight/texture (few performers)	< 0.000
Symphonic scale works	Smaller-scale works	< 0.000
Assertive/masterful	Mild/submissive	< 0.000
Controlled/objective	Sensitive/emotional (with tempo)	< 0.000
Stressed/dramatic	Calm/reflective	< 0.000
Inference that composers are male	Females do not compose	< 0.000

Probability levels are from statistical tests reported above.

**Figure 6:**
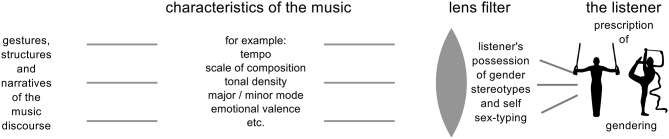
**The lens of gender: Musical properties engender gendering**.

The properties identified here are strongly reminiscent of gender-equivalent polarities reported by Tagg (1989) from responses to TV music, and to those hypothesized by Halstead (1997, p. 239; Table 5 below) and together they indicate that gendering of music is predicated on a complex of stereotypical qualities perceived as representative of masculinity and femininity (Figure 6).

**Table 5:** **Corresponding properties reported by Tagg (1989) and Halstead (1997)**.

**Tagg**		**Halstead**	
**Male**	**Female**	**Male**	**Female**
Fast	Slow	Slow/sluggish	Quick/agile
Active	Passive	Active	Passive
Sudden	Gradual	Large gestures	Small gestures
Strong	Weak	Dominant	Submissive
Staccato	Legato	Striving	Yielding
		Booming sonority	Soft sonority

As Halstead (1997) points out “Although these descriptions are used to refer to different elements of music, they rely on a common understanding of the terms masculine and feminine to underpin their meanings: *feminine* implies weakness, submissiveness, small-scale, whilst *masculine* implies strength, dominance, and large-scale” (p. 233). They reflect commonly accepted stereotypes associating *masculin*e with descriptors such as *authority, power, masterful, controlled, possessing gravity, dramatic* and *feminine* with *passive, mild, calm, submissive to authority, lightness of touch, sensitive.*

These schema act as a perceptual lens, through which incoming music information is filtered and gendering is inferred upon the relative presence or absence of attributes in combination in the schema-filter.

The musical properties identified in the model outlined in Figure 6 represent only those factors thrown into focus by this study, and we do not suggest that they represent a complete list of those that might contribute to a schema; Halstead (1997) hypothesizes a much broader list, suggesting “a whole range of elements which together constitute music, including intensity, pitch structure, form, tonality, duration, instrumentation, timbre, style” that may contribute as part of a network of gender-equivalents (p. 246).

The high significance levels of male-vs.-female attributed musical properties (Table 4) indicate that our listeners made composer-sex resolutions on the basis of these surface features present in the gender-schema lens model. But these features are manifest in the music of both male and female composers, and so did not constitute criteria for differentiation of works by the sex of their composers. Listeners therefore showed no competence in identifying the sex of composers of the extracts they heard, and their attributions to them of gendered masculine and feminine properties can therefore be seen to have been prescriptive.

Interpretation of these grouped factors as evidence of the presence of gendered information in the extracts is further negated by the evidence of the MASCFEM values that showed the extracts from compositions by men to have been perceived as having greater sense of femininity than did compositions by women. As Green (1997b) comments “It is not that there is anything feminine about the inherent meanings, but that the idea of femininity filters our response to them” (p. 100). Halstead (1997) describes this process in a similar way:

“Music seems to be perceived as gendered through the way in which the whole range of elements, which together constitute music (intensity, pitch, structure, form, tonality, duration, instrumentation, timbre, style, and so on) are processed by the individual into a network of gender equivalents” (p. 246).

Evans et al. (2010, p. 176) describe such a process as *self-authorship*, by which an individual categorizes incoming information as gendered according to its conformity to the sets of elements that belong to societal classifications of masculinity or femininity rather than to actual properties of the object, a process explicable under Bem's (1981) gender-schema theory.

With regard to Bem's (1993) proposal that an individual's own self-concept and degree of self sex-typing is influential in the process of gendering, it was not within the scope of this study to consider the self-typology of our listeners, but our results showed no evidence of tendencies for male and female listeners to respond in sex-differentiated ways in their attributions of gendering for the extracts they heard. Insofar as our variable *sex-of-listener* can be expected to at least have some relationship to *sex-type-of-listener*, this element of Bem's theory seems not to have been operative here.

The third hypothesis is therefore accepted, but with the important caveat that the gendered perception understood by listeners was not derived from elements inherent in the data of the music's content, but from the presence of gender stereotyping contributed to the perceptual event by the listener.

The strong correlations between MASCFEM values, composer-sex attributions and the compositional scale and tonal density of musical texture of the works from which the extracts were taken reveals that a view that works of major proportions (symphonies, concerti, choral works) are composed by men, but women are either incapable of attempting large-scale works, or only of sustaining musical invention only briefly, writing only “graceful little songs about spring and the birdies” (Moore, cited in Tick, 1975) is still inherent and, held even among experienced musicians of both sexes.

### Review of measures

The consistent levels of correlation between MASCFEM scale values and other dimensions examined in this study confirm the scale as a valid and useful tool in measurement of gendering and its usefulness in obtaining more detailed data than raw sex-attribution scores.

The remarkable inter-listener consistency shown by the Music Characteristics listeners and the high correlations between the scales themselves (circa *r* = 0.624) indicate their validity as descriptors of music's emotional valence.

## Limitations of the study and issues for further research

### Applicability to other musical genres

This study has focused on music of the classical art music genre; whether its findings are equally applicable to music of other styles, for example pop, jazz, folk, etc. has not been tested. There are several reasons why their wider relevance could not be considered here. Firstly, it was essential that our participants should be expert listeners, sensitive to subtle qualities and elements in the music they heard. Given that experience and discrimination of musical styles, classical versus pop, tends to be singular rather than pluralistic, listeners competent in multiple genres would not be easy to find.

Pop music is substantially vocal and is often strongly biased toward sexual interest. Separation of the gendered verbal boy/girl element from the gestures and structures of the music would be a near impossible achievement for listeners.

In the classical canon, the art work is enshrined in its musical score: adjustment of tempo, dynamics, and nuance are expected of the performer, but the musical text is sacrosanct. Pop, jazz, and folk musical forms are substantially improvisatory, and their sounds are rarely encapsulated in notation in the way that is an essential of art music. Items of pop music are consequently presented in widely differing forms; there are no *authorized versions*. In jazz, the contribution and deviation of the individual are celebrated in the solo *break*.

The identity of the composer is relatively unimportant in non-classical styles: items are not announced as “*It's love, baby” by John Brown, Opus 50, no. 2,”* and the message of the music is overlaid by the influence of the performers. Creation of songs is frequently the product of cooperative contributions among group members of both sexes.

Finally, an issue of pragmatism: the likelihood of creating over-long tasks by sampling multiple musical styles would risk exceeding the willingness and focussed attention of participants.

The wider applicability of our findings to other musical genres would therefore require further research and different experimental approaches.

### Tonal density and instrumental timbre

Tonal weight/density (few performers/instruments—many performers/instruments) has been demonstrated to be an important variable in attribution of gendering, but the tonal character and identity of contributing instruments is an unavoidable correlate of tonal density. It is highly likely, for example, that a composition performed by a flute choir might be judged quite differently from the same work performed by brass or vocal ensembles. Within the limitations of the present study, and the need to keep listening tasks to manageable lengths, it was not possible to evaluate the effect of differing timbral combinations adequately. Research designs focusing on this question would not be difficult to construct, however.

### Gender and self-concept

The interrelationship of gendering and self-concept was not examined in this study. It is possible that degree of gendering imposed by a listener may be conditioned by sex-self type or sexual orientation of the listener. This would be a relevant avenue of investigation.

### Gendered perception of instruments

Although gendered perception of instruments in both adults and children has been evidenced in extensive research, causal factors have not yet been specifically identified (O'Neill, 1997, p. 54) Possibilities include: relation of pitch range of instruments to voice range of listener (Bhatara et al., 2015), position at which the instrument is held (in front of face with face obscured/below face with face visible), size of instrument in relation to physical stature of player, social conditioning, and gender-boundary restriction (Best, 1983; Thorne and Luria, 1986) or, perhaps, all of these.

## Conclusions

Our results indicate that gendered information is not represented in the gestures, structures and narratives of a musical composition. No codes are embedded in music by composers that might operate as hidden signifiers of gender. Any gendered impressions experienced by a listener are imposed onto the incoming musical stimuli subjectively by that listener, contributed from a network of previously established gender schemata, operating at subliminal level, which rely on universal socially acquired stereotypical perceptions of relative characteristics of men and women. Masculinity and femininity are mapped onto the music by the listener.

It has previously been demonstrated that gendering is not imposed on music by the performer (Sergeant and Himonides, 2014); if, as our results demonstrate, gendering is not inherent in musical phenomena themselves, then a necessary conclusion is that gendering originates in the only remaining link in the music communication chain—i.e., by subjective prescription by the listener. In this case, it is possible that the degree of perceived gendering might vary according to the listener's self-concept and degree of sex-self typing.

If, as we have argued, gendering of phenomena and objects is essentially founded on socially agreed gendered behavioral styles (Figure 3), Twenge's evidence (1997) of women's increased endorsement of masculine-stereotyped traits and men's less positive endorsement of masculine-stereotyped traits suggests that differences in gendering are narrowing over time. We may assume therefore that gendered meanings attaching to phenomena such as music will adjust accordingly. If attributed gendered meanings may shift with changing social perceptions, they may not be regarded as inherent properties of phenomena but as subjective dimensions of perception.

Gender schemata operate equally in perceptual processes of male and female listeners: we have found no differences in response behaviors attributable to listener sex for any of the variables tested, and no evidence of own-sex insights. Although gendering of music is certainly a reality, it is not a property of music, but of its listener: “Masculinity and femininity exist only in the mind of the perceiver” (Bem, 1987) and in the social structures that shape that mind. We therefore propose that study of gendering of music is a legitimate field of inquiry within the discipline of Psychology, but not of Musicology.

## Author contributions

Dr DS and Dr EH claim authorship of this manuscript and also the copyright of the research work that this manuscript reports.

### Conflict of interest statement

The authors declare that the research was conducted in the absence of any commercial or financial relationships that could be construed as a potential conflict of interest.

## Footnotes

^1^The device of adopting masculine names did not greatly advance the fame of the three women poets, as only two copies of their volume were sold!
