# Supplementary material for: Gender and Music Composition: A Study of Music, and the Gendering of Meanings
Source: Front Psychol. 2016 Mar 31;7:411. doi: 10.3389/fpsyg.2016.00411 (PMC4815278; doi:10.3389/fpsyg.2016.00411)
Supplement: Supplementary file 1 [file DataSheet1.docx]

**Appendix**

**List of extracts comprising the Listening Sequence**

**(N.B. The order of presentation of extracts was randomized into a unique order for each of the 71 listeners)**

Extract no.

| 1 | Reynaldo Hahn (1884–1947) | Chanson d’Automne | solo voice and piano |
| --- | --- | --- | --- |
| 2 | Amy Beach (1867–1944) | The year’s at the spring | solo voice and piano |
| 3 | Roger Quilter (1877–1963) | Spring is at the door | solo voice and piano |
| 4 | John Ireland (187 9–1962) | The peaceful western wind | choral ensmble |
| 5 | Fanny Mendelssohn (1805–1847) | Chorus from cantata ‘Job’ | chorus and orchestra |
| 6 | Josephine Lang (1815–1880) | Der Winter | solo voice and piano |
| 7 | Barbara Strozzi (1619–1664) | Al batitor di Bronzo | 2 solo voices and continuo |
| 8 | Lennox Berkeley (1903–1989) | A Dinner Engagement: scene 1. The Kitchen | 2 solo voices and piano |
| 9 | Cyril Scott (1879–1970) | Rainbow Trout | piano solo |
| 10 | Ethel Smyth (1858–1944) | Fugue in C major | piano solo |
| 11 | Germaine Tailleferre (1892–1983) | Pastorale in D min | piano solo |
| 12 | Florence Beatrice Price (1887–1953) | Suite no 1: Fantasy | organ solo |
| 13 | Clara Schumann (1819–1895) | Scherzo in D min | piano solo |
| 14 | Elisabeth Claude Jacquet de la Guerre (1665–1729) | Tocade | harpsichord solo |
| 15 | George Enescu (1881–1955) | Sonata no 1 F# min Presto vivace | piano solo |
| 16 | Hanns Jelinek (1901–1969) | Charackterstuck | piano solo |
| 17 | Nicolai Medtner (1880–1961) | Arabesque no 7: Idyll, Allegro tranquillo et dolce | piano solo |
| 18 | Christian Sinding (1866–1941) | Con fuoco | piano solo |
| 19 | Louise Farrenc (1804–1875) | Piano qtet A min.: Scherzo | string quartet and piano |
| 20 | Louise Farrenc (1804–1875) | Clarinet trio Eb major Finale: Allegro | clarinet, violin and ‘cello |
| 21 | Ernest Chausson (1855–1899) | Piano trio G min: Andante | piano, violin and ‘cello |
| 22 | Elfrida Andrée (1841–1929) | Piano qtet A min. Allegro molto moderato | string quartet and piano: |
| 23 | Juilius Rontgen (1855–1932) | Oboe sonata: Poco animato | oboe and piano |
| 24 | Germaine Tailleferre (1892–1983) | Forlane | flute and piano |
| 25 | Thomas Dunhill (1877–1946) | Phantasy Suite Allegro con fuoco | clarinet and piano |
| 26 | Thomas Dunhill (1877–1946) | Lyric Suite Nocturne: Andante con animato | bassoon and piano |
| 27 | Elisabeth Claude Jacquet de la Guerre (1665–1729) | Violin sonata F major: Presto | violin and continuo |
| 28 | Francesco Veracini (1690–1768) | Violin sonata A minor: Allegro | violin and piano |
| 29 | Nicola LeFanu (1947– ) | Prelude for Orchestra, no 2 | chamber orchestra |
| 30 | Dag Wiren (1905–1986) | Suite for Strings: Allegro molto | string orchestra |
| 31 | Louise Farrenc (1804–1875) | Symphony no 1. first movement: Allegro | symphony orchestra |
| 32 | Max Reger (1873–1916) | Romantic Suite: Notturno | symphony orchestra |
| 33 | Clara Schumann (1819–1895) | Piano concerto in, A minor: First movement: Allegro maestoso | piano and orchestra |
| 34 | Ernest John Moeran (1894–1950) | Rhapsody for piano and orchestra | piano and orchestra |
| 35 | William Sterndale Bennett (1816–1875) | Piano concerto in F minor: First movement: Poco agitato | piano and orchestra |
| 36 | Amy Beach (1867–1944) | Piano concerto in. C# minor: Second movement: Largo | piano and orchestra |
